# Effects of a vildagliptin/metformin combination on markers of atherosclerosis, thrombosis, and inflammation in diabetic patients with coronary artery disease

**DOI:** 10.1186/1475-2840-11-60

**Published:** 2012-06-06

**Authors:** Robert Klempfner, Jonathan Leor, Alexander Tenenbaum, Enrique Z Fisman, Ilan Goldenberg

**Affiliations:** 1 Cardiac Rehabilitation Institute, Leviev Heart Center, Sheba Medical Center, 52621, Tel Hashomer, Israel; 2 Cardiac Research Institute, Leviev Heart Center, Sheba Medical Center, 52621, Tel Hashomer, Israel; 3 Sackler Faculty of Medicine, Tel Aviv University, 69978, Ramat Aviv, Israel; 4 Cardiovascular Diabetology Research Foundation, 58484, Holon, Israel

**Keywords:** Type 2 diabetes, Vildagliptin, Metformin, Atherosclerosis, Inflammation, Interleukin-6, TNF, Atherothrombosis, Adiponectin, MMP-9, hs-CRP

## Abstract

**Background:**

Diabetic patients present with an accelerated atherosclerotic process and an increased risk for future cardiovascular events. In addition to the risk imposed by the disease itself, pharmacological treatment adds also a sizable risk, especially if certain classes of antidiabetic drugs are employed. Animal evidence indicates that dipeptidyl peptidase-4 inhibitors have anti-atherosclerotic effects, yet clinical data are scarcely available.

**Design:**

We plan to prospectively investigate the effects of dipeptidyl peptidase-4 inhibition with vildagliptin on a number of atherothrombotic markers and adipokines in patients with proven atherosclerosis and type 2 diabetes. The selected markers are: interleukin-6, high sensitivity C reactive protein, interleukin 1-beta, total adiponectin levels, matrix metallo-proteinase 9 and platelet reactivity testing. Sixty eligible patients will be randomized in a 2:1 ratio to vildagliptin/metformin or metformin only treatment, for a 3-month duration treatment. Blood sampling for the proposed investigations will be taken at enrollment and immediately after completion of the study period.

**Discussion:**

Demonstrating antiatherothrombotic properties of dipeptidyl peptidase-4 inhibitors on proven markers is of substantial clinical significance. Coupled with their proven good safety profile these findings could translate into a significant clinical benefit.

## Background

Patients with ischemic heart disease and diabetes are at a particularly high risk for the recurrence of cardiovascular events. Conversely, certain classes of oral antidiabetic medications have been shown to cause hypoglycemia as well as adverse cardiovascular effects [1-3]. Diabetes induces complex vascular changes, promoting accelerated atherosclerosis and hypercoagulability, as can be assessed indirectly by a number of markers. Principal perturbations include endothelial dysfunction, increased inflammatory plaque infiltration, adhesion molecule over-expression and adverse effects of circulating fatty acids and advanced glycosylation end products.

Animal studies have suggested numerous beneficial antiatherosclerotic changes of dipeptidyl peptidase-4 inhibitors (DPP4i), well beyond the effects on blood glucose alone [4,5]. Additionally, antiremodeling effects are proposed [6]. However, this feature has not been established in a clinical setting. Concomitant treatment with a DPP4i and metformin may offer an attractive glycemic reduction modality with synergistic mechanism of action while exerting additional vascular protective benefits. Reduction of inflammatory marker levels is of great clinical importance and has been shown to correlate with reduction in significant clinical events. Therefore, in the present study we plan to focus on possible anti inflammatory and atherothrombotic protective effects of DPP4i in a clinical setting.

Key representative markers for the present study are chosen in order to correctly represent alterations in: inflammation (hs-CRP), risk of atherosclerotic plaque rupture and matrix turnover (MMP-9), and platelet reactivity (aggregability tests). These markers are further detailed:

### Interleukin 6 (IL-6)

This established inflammatory marker has been shown to be increased in individuals with coronary artery disease [7,8]. Furthermore, diabetes has been referred to as a chronic inflammatory state. Therefore, a reduction in inflammatory markers in this high-risk population is likely to correlate with a corresponding reduction in the risk for atherothrombotic events.

### High sensitivity C-reactive protein (hs-CRP)

Marker of inflammation with a strong correlation with cardiovascular events even in normolipemic population [9]. Reduction of hs-CRP has been demonstrated with vildagliptin-pioglitazone combination, but data from patients with cardiovascular disease and vildagliptin-metformin combination are lacking [10].

### Platelet reactivity testing

Platelets are hyper-reactive in diabetic patients, and this heightened activity is closely linked to vascular events [11,12]. Perturbations in both structure and function have been described [13,14]. Reduction in reactivity is a viable surrogate of reduced thrombogenic milieu [15]. However, currently there are no human data on the effects of DPP4i on platelet function. We hypothesize that a combined vildagliptin-metformin therapy will be associated with a greater reduction in platelet reactivity as compared with metformin monotherapy.

### Adiponectin

Hormone with regulatory metabolic function secreted from the adipose tissue. Reduced levels of adiponectin were shown to be associated with obesity, metabolic syndrome, and diabetes, and to promote the atherosclerotic process [16-18]. Higher levels have been found to be protective [18,19]. Reduction in adiponectin levels induced by fatty diet has recently been shown to be corrected by DPP4i in mice [4], yet the effect in diabetic humans is unknown.

### Matrix metallo-proteinases 9 (MMP-9)

The MMPs are a large family of zinc-dependent, extra-cellularly acting endo-peptidases. Substrates of MMPs are proteins of the extracellular matrix and adhesion proteins. Patients with coronary artery disease were recently shown to have increased levels of MMP-9 [20]. A higher MMP-9 level was also shown to correlate with coronary artery ectasia [21], and to predict increased mortality in patients with coronary artery disease [22]. Accordingly, we hypothesize that treatment with combined vildagliptin-metformin therapy will be associated with significantly greater reductions in MMP-9 levels as compared with metformin monotherapy.

The effect of DPP4i on the above-mentioned parameters has not been studied in humans. Accordingly, the demonstration of significant improvements in markers of atherothrombosis and inflammation in high-risk diabetic patients is of great clinical importance and novelty that may be employed for the reduction of major cardiovascular events in this population.

## Study design

### Study purpose

To demonstrate that combined vildagliptin-metformin therapy is associated with clinically significant reductions in biological markers of inflammation, pro-thrombogenicity, and atherosclerosis as compared to metformin monotherapy in a population of diabetic patients with coronary artery disease who undergo cardiac rehabilitation.

The prespecified established biological markers of inflammation, pro-thrombogenicity, and atherosclerosis will include: interleukin-6 (IL-6 – primary biological marker), hs-CRP, platelet reactivity testing, MMP-9, interleukin 1 beta (IL-1 beta) and adiponectin levels.

Findings from this analysis will demonstrate the pleiotropic effects of DPP4i in a clinical setting, in addition to its established antiglycemic effects.

### Specific aims

 2.1. Primary Aim: Demonstration of a significant (≥ 20%) reduction in the serum levels of interleukin 6 (IL-6)

 2.2. Secondary Aims:

 Improvement in other markers of atherothrombosis and inflammation:

 High sensitivity C-reactive protein (hs-CRP),

 Platelet reactivity

 Adiponectin levels

 IL-1 beta

 Matrix metallo-proteinase 9 (MMP-9)

 Additional exploratory markers including: IL-1 alpha, IL-17, TNF-alpha, MCP-1, monocyte subsets by FACS 

 Safety from hypoglycemic events

 Reduction in glycosylated hemoglobin (Hb A1c)

 Weight reduction

## Investigational plan

### Rationale of study design

**Figure 1:**
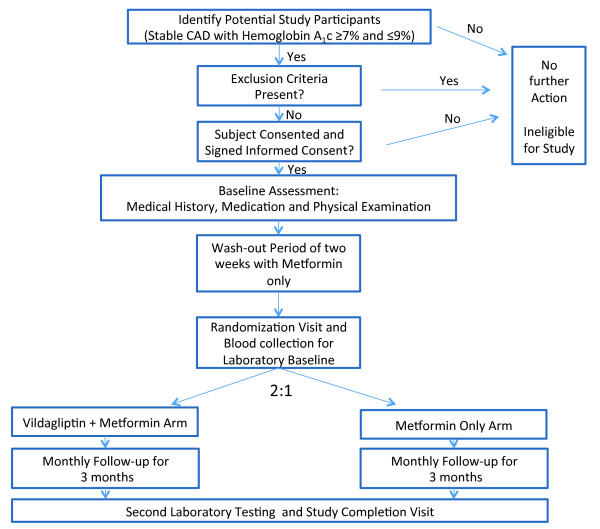
Figure 1 Study design and flow.

The study is designed as a single-center, randomized, non-blinded, clinical trial to provide evidence on the effects of vildagliptin on key biomarkers of atherothrombosis and inflammation. We plan to prospectively enroll 60 patients with proven coronary artery disease and randomize them in a 2:1 ratio to either vildagliptin-metformin therapy (n = 40) or metformin therapy (n = 20). Study design and flow are presented in Figure 1.

### Rationale of dose, duration of treatment

Vildagliptin-metformin combination is approved by the ministries of health and is commercially available in Israel and Europe. It has been shown to induce significantly less hypoglycemic events and therefore lower sympathetic activation detrimental for cardiac patients. The effects of vildagliptin-metformin combination vs. metformin monotherapy will be assessed 3-months after initiation of therapy. The short duration of the study and frequent follow-up visits with weekly interaction with study personnel will ensure the safe conduct of this trial and improve participant adherence. The 3-month duration has been shown in previous marker centered studies to be sufficiently long to induce the anti-atherothrombotic and anti-atherosclerotic changes. Metformin has been chosen for the comparison group, for the following reasons: 1) the drug was shown to be one of the most efficacious therapeutic options available [23,24]; 2) the clinical experience with the drug is extensive; and 3) it is a component of the comparison group vildagliptin-metformin combination.

### Study population

· Participants eligible for this trial will include male and non-child-bearing potential female patients age 21 years and older who have (a) documented coronary artery disease > 30 day; and (b) evidence of suboptimal type II diabetes control on the basis of Hb A1c ≥7.0%, despite the use of oral antidiabetic monotherapy. Standard of care secondary prevention for coronary artery disease background therapy will include, but will not be limited to, lipid lowering, antihypertensive, beta blockers, and antiplatelet therapy, as appropriate and in accordance to current guidelines.

### Inclusion criteria

· Type 2 diabetes mellitus on oral mono-therapy or diet only treatment

· Stable documented ischemic heart disease (>30 days post AMI, CABG or PCI)

· Suboptimal Hb A1c as defined ≥7.0%

· Age > 21

· Life expectancy >1 year

### Exclusion criteria

· Significant renal impairment (creatinine ≥1.4 mg\dL females or ≥1.5 mg\dL males)

· Planned coronary intervention or planed surgical intervention (PCI or CABG)

· Planned surgical intervention

· Recent (<30 day) acute coronary syndrome (ACS)

· Hypersensitivity to either of the study drug components

· History of lactic acidosis

· Type I diabetes

· Current Hb A1c >9%

· Current Insulin treatment

· Active treatment with GLP-1 or other DPP4i medication

· Hepatic impairment or ALT\AST elevations beyond X2 upper normal limit or known hepatic failure

· Inability to comply with study protocol

· Active malignancy other than basal cell carcinoma

· Clinically advanced congestive heart failure - NYHA III-IV

· Severe left ventricular dysfunction (LVEF < 30%) with NYHA II or any NYHA class with documented recent heart failure decompensation (<3 months)

· Severe stable cardiac angina CCS III – IV or Unstable angina

· Chronic inflammation (i.e. inflammatory bowel disease, lupus, inflammatory arthritis, rheumatoid arthritis) or chronic infection (i.e. chronic diabetic foot infection)

· Pregnancy, lactation or child-bearing potential

### Baseline clinical evaluation

At baseline patients will undergo detailed assessment including: 1) focused medical history, past cardiovascular events and key clinical findings, 2) body mass index (BMI) measurements, 3) physical examination and medication history. Based on current medication patients will be initiate a wash-out period of 2-weeks as described below.

### Prior antidiabetic medication washout

Eligible patients (Hb A1c ≥7% and ≤ 9%) who receive current anti-diabetic monotherapy (not including metformin or a DPP4i) who consent to participate in the study will initially receive substituted antidiabetic treatment with metformin. Prespecified substitution of oral antidiabetic monotherapy is permitted if clinically reasonable and safe. A washout period of two weeks will take place prior to randomization. During this period, treatment with open label metformin will be carried out with blood glucose monitored regularly. Initial dose will be 850 mg once daily, with a dose increase to a maximum of 850 mg TID stabilization to a target of fasting glucose ≤130 mg/dL. For patients eligible for the study who receive current treatment with metformin monotherapy, a dose increase will also be allowed to a maximum of 850 mg TID aiming for stabilization to a target of fasting glucose ≤130 mg/dL.

### Stabilization of oral anti-diabetic therapy for eligible patients not on previous therapy

Eligible patients (HbA1C ≥7% and < 9%) not on current antidiabetic therapy who will consent to participate on the study will be initiated with open-label metformin monotherapy for a period of two weeks prior to randomization. During this period, treatment with metformin will be carried out with blood glucose monitored regularly. Initial dose will be 850 mg once daily, with a dose increase to a maximum of 850 mg TID until stabilization to a target of fasting glucose <130 mg/dL.

### Randomization

Randomization will be carried out following a 2-week period of stabilization on metformin mono-therapy as described above. Patients will be randomized in a 2:1 ratio to open label treatment with vildagliptin-metformin therapy or metformin mono-therapy. This study will be non-blinded to patients and treating personnel. All laboratory tests, the major study endpoints, will be performed by professionals blinded to treatment allocation. An interim analysis will be performed after the recruiting 50% of proposed study participants.

### Treatment

The entire study group will be actively treated orally with either metformin (control group) or metformin-vildagliptin combination (intervention group), once or twice daily. No placebo medication is used in this study. The study is open-labeled. Endpoints are mostly laboratory ones, so the un-blinded design will have a minimal effect on the validity of the obtained results.

Treatment will be initiated as follows: (1) in the control arm, metformin dose will be based on the treatment prescribed at the end of the 2 week pre-randomization period; and (2) in the investigational arm, metformin will be replaced with combined metformin-vildagliptin treatment with an initial dose of once daily. Titration to the three times a day is permitted in accordance to investigators clinical judgment in the control arm, while in the metformin-vildagliptin combination, maximal dose will be twice daily.

Study patients and other treating physicians will be asked not to alter antidiabetic medication unless there is justifiable clinical urgency. As statin have proven anti-inflammatory and diverse pleotropic properties, statin type and dose will be recommended to remain fixed during the 3-month study period, unless otherwise clinically indicated.

### Medication titration

Study design permits, based on blood glucose measurements and additional lab test if deemed necessary, modification of the medication dose with the goal of fasting glucose ≤ 130 mg/dl. In the control arm up-titration will be carried out with metformin, and in the treatment arm up-titration will be carried out with vildagliptin-metformin combination. If control of blood glucose cannot be achieved after maximal drug titration and there are repeated (>3) measurements of FPG > 300 mg/dl after nutritional consultation and exercise, the study investigator is encouraged to add antidiabetic treatment according to medical judgment and national guidelines. Deviations from study protocol are to be recorded in detail on appropriate eCRF. With the exception of severe exacerbations in glucose homeostasis noted earlier, all effort is to be made to maintain a stable treatment plan in coordination with the family physician. Patient’s adherence to study medication will be evaluated by pill-count at the follow-up visits.

### Follow-up visits

All patients will be invited for monthly follow-up visits with study coordinators. During these visits we plan to monitor both clinical and adverse events, verify medication compliance and evaluate any hypoglycemic events. Trained medical personnel will verify the home monitoring glucose journal. Changes in weight and in drug regiment will be recorded. At the completion of 3-months blood will be drawn for laboratory testing as done at baseline. Blood samples will not contain identifying information and all tests are to be performed in a blinded fashion.

### Statistical methods and power calculation

Sample size justification: We expect to show an improvement (defined as >20% biomarker reduction between the baseline and 3-month assessment) in ≥3 of the prespecified biomarkers that comprise the primary end point in at least 50% of the patients randomized to combined vildagliptin-metformin therapy and in <10% of the patients randomized to metformin mono-therapy. The proposed sample size is calculated to demonstrate a significant improvement in the intervention group compared to the control group with at least 90% power and a two-sided 5% type 1 error. These requirements are met with a sample size of 60 patients and a 2:1 randomization design: 40 patients in the combined vildagliptin-metformin intervention group and 20 in the metformin monotherapy control group.

## Discussion

Treatment of diabetes is more than the reduction of blood glucose [24,25]. Patients with glycosylated hemoglobin within guidelines recommended values have a substantial residual risk compared to the healthy population. As atherosclerosis is the leading cause of death and morbidity in the diabetic population, interventions that potentially reduce this risk are of crucial importance. Statins have been shown to substantially reduce the risk of MACE in diabetic patients with pleiotropic effects beyond the reduction of LDL [26-28]. Animal models found intriguing antiatherosclerotic influences that could translate into a substantial clinical benefit [4].

The design of this study serves two important roles: first, the short duration will permit meticulous follow-up, and adherence to protocol. Secondly, it will enhance safety and minimally interfere with regular patient management. In order to reduce interference from concomitant medication changes, especially statins, we plan to enroll patients with a stable drug regiment that is in accordance to national guidelines. We will encourage the managing physician to refrain from drug or dose changes within the short study duration unless deemed necessary. We did not deem it important to blind treatment as all study end-points are laboratory ones, performed on de-identified blood samples by personnel blinded to patients data or study allocation. This open-labeled design will also enhance patient safety. Cardiovascular safety of antidiabetic medication is of paramount importance and has been under recent FDA scrutiny [29]. A number of hypoglycemic drugs, especially sulfonylureas, have been associated with significant hypoglycemia and adverse events induced by sympathetic activation. Activation of the sympathetic system has numerous implications, including surges of heart rate, blood pressure but also proinflammatory and procoagulant effects. This partially explains the increased cardiovascular adverse events noted with these drugs. DPP4i have a good safety profile [30,31]. A vildagliptin-metformin combination has the potential to reduce the rate of hypoglycemic events. Notably, the anti-inflammatory effect of DPP4i may be partially explained by lack of repetitive sympathetic activations. These hypothesized protective effects will be evaluated in the clinical setting of the proposed study.

## Abbreviations

ACS: Acute coronary syndrome; CABG: Coronary bypass artery grafting; DM: Diabetes mellitus; DPP4i: Dipeptidyl peptidase-4 inhibitors; EST: Exercise stress test; FPG: Fasting plasma glucose; Hb A1c: Glycosylated hemoglobin; Hs-CRP: High sensitivity C reactive protein; IL: Interleukin; LVEF: Left ventricular ejection fraction; LDL: Low density lipoprotein; MCP: Macrophage chemotactic protein; MACE: Major adverse cardiovascular events; MMP-9: Matrix metallo-proteinase 9; PCI: Percutaneous coronary intervention.

## Competing interests

The authors declare that they have no competing interests.

## Authors’ contributions

IG and RK conceived the study, and participated in its design, coordination and drafted the manuscript with EF and AT. JL participated in the design of the study. All authors read and approved the final manuscript.
